# Supplementary material for: Interface control by homoepitaxial growth in pulsed laser deposited iron chalcogenide thin films
Source: Sci Rep. 2015 Nov 9;5:16334. doi: 10.1038/srep16334 (PMC4637838; doi:10.1038/srep16334)
Supplement: Supplementary Information [file srep16334-s1.pdf]

Supplementary Information

Interface control by homoepitaxial growth in pulsed laser deposited iron chalcogenide thin films

Sebastian Molatta[1,2,3,*], Silvia Haind[3,4], Sascha Trommler[2,3], Michael Schulze[2,3], Sabine Wurmehl[2,3], Ruben Hühne[3]

[1]Dresden High Magnetic Field Laboratory (HLD-EMFL), Helmholtz-Zentrum Dresden-Rossendorf, D-01314 Dresden, Germany
[2]Dresden University of Technology, Department of Physics, D-01062 Dresden, Germany
[3]Leibniz-Institute for Solid State and Materials Research (IFW) Dresden, Helmholtzstraße 20, D-01069 Dresden, Germany
[4]Physikalisches Institut, Universität Tübingen, Auf der Morgenstelle 14, D-72076 Tübingen, Germany
[*]corresponding author's e-mail address: s.molatta@hzdr.de

S I XRD of unseeded film at high deposition temperature

In Fig. S 1 the XRD θ–2θ-scans of films grown directly on MgO at T_D's of 530 °C and 300 °C are shown. Besides *c*-axis grown components of the film with $T_D = 530$ °C ((00*l*)-reflections) there are additional *out-of-plane* orientations observable. Peaks of the (*h*0*l*) as well as the (*hkl*) orientation are found [Fig. S 1(a)]. With decreasing T_D the intensity of the peaks for orientations different from (00*l*) decreases rapidly. The XRD θ–2θ-scan of the film with $T_D = 300$ °C shows exclusively *c*-axis oriention *out-of-plane* [Fig. S 1(b)].

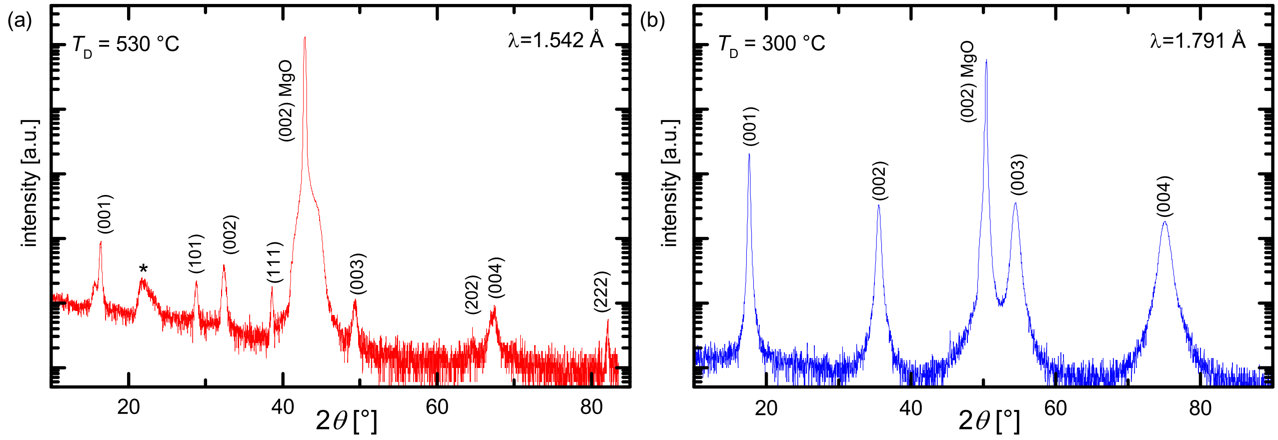

Figure S 1. XRD θ–2θ-scan of $FeSe_{1-x}Te_x$ thin films grown on MgO at (a) $T_D = 530$ °C and (b) $T_D = 300$ °C. The $\star$ indicates the $\lambda/2$-peak of MgO.

S II In-plane texture: Comparison between unseeded and seeded films on MgO

Evaluating the FWHM of the peaks of the *in-plane* orientation of the temperature series [Fig. S 2] shows a strong increase of $\Delta\phi$ for both texture components for the thin films without seed layer for decreasing T_{D}. In contrast to that, the $\Delta\phi$ values of thin films with seed layer stay more or less constant with decreasing deposition temperature.

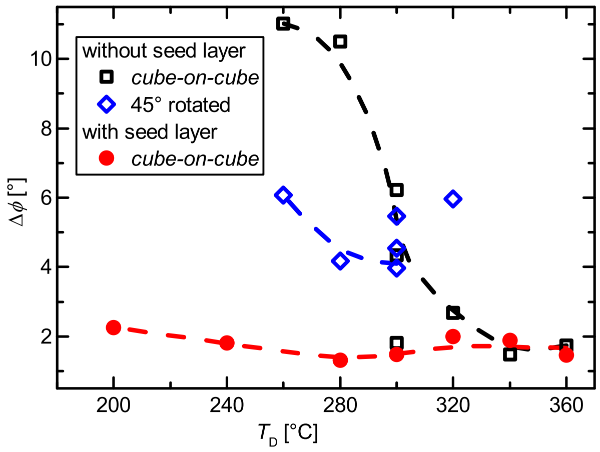

Figure S 2. $\Delta\phi$ of the texture components of the samples without and with seed layer in dependence of T_{D}.

S III Resistance measurements of films without seed layer

From the resistance measurements [Fig. S 3] $T_{\mathrm{c,90}}$ and ΔT_{c} for the thin films without seed layer are obtained. ΔT_{c} is calculated from the temperature where the resistance reaches 90% of the normal state resistance minus the temperature where the resistance reaches 10% of the normal state resistance.

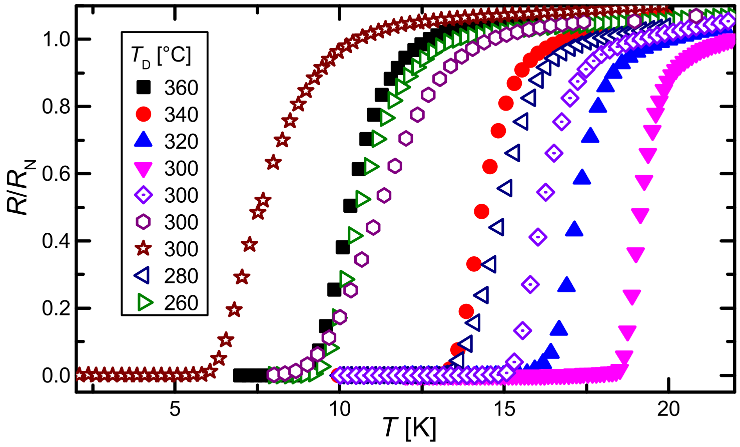

Figure S 3. Temperature dependence of the normalized resistance ($R_{\mathrm{N}} = R(100\%)$) for thin films without seed layer.

S IV Comparison of the surface morphology

The surface morphology of a sample without seed layer [Fig. S 4 (a) $T_D = 300\,°C$, $\tau = 1$] and a sample with seed layer [Fig. S 4 (b) $T_D = 240\,°C$] was evaluated with AFM. The rms-values of the samples show an improvement of the surface roughness by a factor of 2.

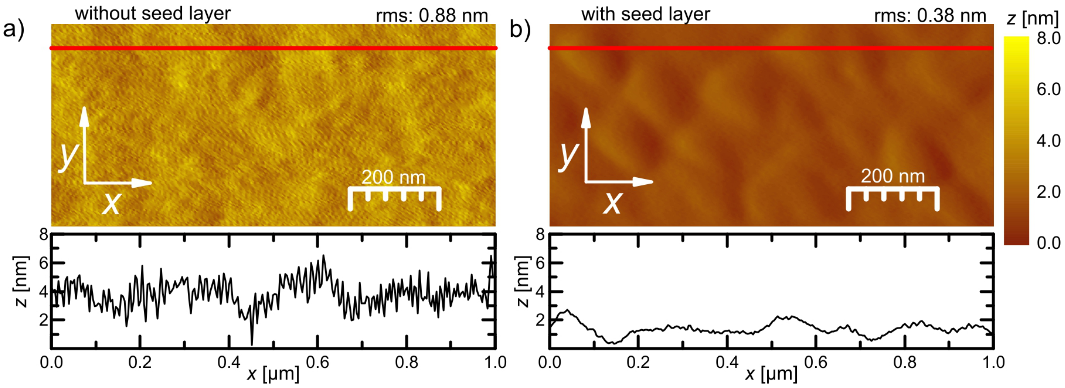

Figure S 4. AFM images of a thin film grown without seed layer [(a) $T_D = 300\,°C$, $\tau = 1$] and a film on the seed layer [(b) $T_D = 240\,°C$]. The red lines mark the position of the profiles below.
